# Propofol Requirement and EEG Alpha Band Power During General Anesthesia Provide Complementary Views on Preoperative Cognitive Decline

**DOI:** 10.3389/fnagi.2020.593320

**Published:** 2020-11-27

**Authors:** Cyril Touchard, Jérôme Cartailler, Charlotte Levé, José Serrano, David Sabbagh, Elsa Manquat, Jona Joachim, Joaquim Mateo, Etienne Gayat, Denis Engemann, Fabrice Vallée

**Affiliations:** ^1^Department of Anesthesiology and Intensive Care, Lariboisière – Saint Louis Hospitals, Paris, France; ^2^Inserm, UMRS-942, Paris Diderot University, Paris, France; ^3^Université Paris-Saclay, Inria, CEA Palaiseau, France; ^4^Department of Neurology, Max Planck Institute for Human Cognitive and Brain Sciences, Leipzig, Germany

**Keywords:** EEG signal, general anesthesia (GA), brain age, alpha band power, cognitive decline and dementia

## Abstract

**Background:** Although cognitive decline (CD) is associated with increased post-operative morbidity and mortality, routinely screening patients remains difficult. The main objective of this prospective study is to use the EEG response to a Propofol-based general anesthesia (GA) to reveal CD.

**Methods:** 42 patients with collected EEG and Propofol target concentration infusion (TCI) during GA had a preoperative cognitive assessment using MoCA. We evaluated the performance of three variables to detect CD (MoCA < 25 points): age, Propofol requirement to induce unconsciousness (TCI at SEF_95_: 8–13 Hz) and the frontal alpha band power (AP at SEF_95_: 8–13 Hz).

**Results:** The 17 patients (40%) with CD were significantly older (*p* < 0.001), had lower TCI (*p* < 0.001), and AP (*p* < 0.001). We found using logistic models that TCI and AP were the best set of variables associated with CD (AUC: 0.89) and performed better than age (*p* < 0.05). Propofol TCI had a greater impact on CD probability compared to AP, although both were complementary in detecting CD.

**Conclusion:** TCI and AP contribute additively to reveal patient with preoperative cognitive decline. Further research on post-operative cognitive trajectory are necessary to confirm the interest of intra operative variables in addition or as a substitute to cognitive evaluation.

## Introduction

Cognitive Decline (CD) is characterized by an impairment or a gradual weakening in cognitive functions such as memory, language, or judgment (American Psychiatric Association, 2013; Purdon, P. L., et al., 2015). Besides being common among elderly people, it is also associated with an increased risk to develop postoperative neurocognitive disorders (Greene et al., 2009; Lee et al., 2011; Silbert et al., 2015). These complications are linked to poor long-term outcomes including increased mortality, loss of autonomy and dementia (Inouye et al., 1998; Sprung et al., 2017). The estimated incidence-rates of preoperative CD might affect at least 24% of people over an age of 65 old (Culley et al., 2017). The increase in surgical procedures among the elderly, as a results of ageing population, has recently motivated the medical community to administer rapid neurocognitive tests prior to surgical intervention (Berger et al., 2018). In practice, such assessments are time-consuming and come with several pitfalls related to test administration. Moreover, stress and pain related to surgical procedure may systematically distort the measurements. Can the perioperative cognitive risk be objectively assessed without recourse to a patient or operator dependent scale?

Aging is a heterogeneous process leading to strongly individualized trajectories in cognitive functioning and health. Retrieving the date of birth from the patient's passport to determine cognitive risk is therefore not sufficient. This longstanding problem has early-on stressed the difference between chronological age and biological age (Chown, Dirken and Siegler in the '60s to '80s) and therefore the importance of research on specific measures of precocious and accelerated aging. In the past decade, the advent of machine learning in neuroimaging and biomedical research has led to burgeoning interest in proxy measures for individual aging. One such measure is the brain age, which is defined as the difference between the passport age and the age algorithmically predicted from population-level brain images (Dosenbach et al., 2010; Cole et al., 2019). Elevated brain age has been linked to various facets of cognitive dysfunction, morbidity and mortality (Liem et al., 2017; Cole et al., 2019). Recent studies suggest that brain age prediction can be enhanced by combining several neuroimaging modalities and found that electrophysiology adds unique information on aging (Liem et al., 2017; Engemann et al., 2020). First studies have demonstrated robust estimation of brain age from high-density EEG data (Sabbagh et al., 2020) and sleep EEG (Sun et al., 2019). However, this framework has been rarely applied outside of the laboratory. The simplification of the collection, the non-invasive character, the acceptance by the patient and the least cost are all criteria that could allow the optimization and generalization of the concept.

The EEG recorded during general anesthesia might provide a unique window on individual aging. During the intraoperative period, a systematic monitoring setup provides critical information on vital signals in real time. Among the per-operative arsenal used for patient monitoring, the EEG signal is often the method of choice to evaluate the depth of the sedation and subsequently adjust drug-administration. Some authors proposed a new complementary use of peroperative EEG aimed at predicting CD, or even more generally peri operative neurocognitive disorder, using patterns such as the intra operative Burst Suppression (BS) (Wildes et al., 2019). Recently, the decrease of power spectral density in the alpha band (8–13Hz), collected on the frontal EEG under general anesthesia has been associated not only to chronological age (Purdon et al., 2015) but also to preoperative CD (Koch et al., 2019). On the other hand, it is also recognized that the effective dosage of hypnotics under general anesthesia decreases with age (Schnider model and MAC) but also with preoperative cognitive status (Laalou et al., 2010). We previously presented evidence for an important association of sedation levels and EEG derived variables (Propofol requirement and Alpha band power) with chronological age and BS (Touchard et al., 2019). Yet, the precise relationship between these two variables and CD is still unclear. As relevant large-scale data necessary for machine learning is not available at this point, here we developed a classical statistical model to investigate potential biomarkers of cognitive decline based on standard monitoring data from general anesthesia.

The objective of this present study is to evaluate the relationship between intraoperative EEG, especially the alpha band power, effective Propofol dosage and the pre-operative CD evaluated using the Montreal Cognitive Assessment (MoCA). We propose an intraoperative model assessing indirectly cognitive decline under general anesthesia that we termed HELP: **H**ypnotic requirement and **E**EG signa**L P**ower.

## Methods

### Patient Selection and Intra Operative Data Collection

Between November 2018 and May 2019, patients eligible for interventional neuroradiology or orthopedic surgery performed under general anesthesia were selected to participate in this prospective, observational, mono-centric study. Inclusion criteria were an elective surgery, a Propofol-based Total IntraVenous Anesthesia (TIVA), a French-speaking and adult (>18yo.) patient. Pregnant women, patients sedated under mechanical ventilation at the time of their management or with a BMI > 35 kg/m2 were excluded (TCI pharmacological model not applicable).

Frontal EEG data (Fp1, Fp2, F7, F8, a ground electrode at Fpz and a reference electrode 1 cm above Fpz) were recorded using the Sedline brain function monitor (Masimo Corporation©, Irvine, California, USA). The sampling frequency of analyzable signal was 179 Hz. The intra operative EEG signal was not collected if the electrode impedance was >5 kΩ. Data collected from standard monitoring included: Pulse Oxygen Saturation (SpO2), Heart Rate (HR), Systolic (SBP), Diastolic (DBP) Blood Pressure and Mean Arterial Pressure (MAP), temperature, Expired CO2 Fraction (EtCO2). Patient demographics were collected during the medical consultation with the anesthesiologist. This study has been approved by the SRLF (Société de réanimation de la langue française) Ethics Advisory Committee 11–356. To participate, all volunteers had to provide an oral consent before any study-related activities.

### Anesthetic Protocol

General anesthesia was induced using total intravenous anesthesia (TIVA) (Absalom and Mason, 2017), with first a standardized administration of opioid agent, then Propofol. For neuroradiology procedures, analgesia was provided using remifentanil while boluses of sufentanil were administered every hour for orthopedic surgery patients. The protocol required a standardized administration for all patients, remifentanil was administered according to the Minto model between 2.5 and 3.5 ng/ml and sufentanil administered in iterative bolus doses between 0.2 and 0.3 μg/kg/hour. An instruction was given to the anesthesiologist in charge to maintain the SEF_95_ within 8 and 13 Hz after the oral tracheal intubation by modulating Propofol TCI. All patients were intubated after muscular relaxation obtained with atracurium besilate injection (0.5 mg/kg). They were mechanically ventilated with a tidal volume of 6–8 ml/kg and a with a respiratory rate adapted to obtain an EtCO_2_ between 35 and 38 mmHg. The administration of fluids and vasoconstrictors was left to the discretion of the anesthesiologist present in the operating room. The mean arterial pressure was strictly controlled to stay at or above 70 mmHg and temperature controlled above 36°C.

### Cognitive Assessment by the Montreal Cognitive Assessment (MoCA)

Cognitive functions were evaluated using the MoCA method 1 day before (D-1) the surgical intervention. We defined the cognitive decline (CD) based on a D-1 MoCA score <25 points. Patients with a cognitive decline where designate by CD+, CD– otherwise. Two anesthetists and one anesthetist nurse, trained to administer, interpret and score the MoCA tests, carried out all administrations. Prior to each evaluation, we checked for the absence of a significant pain that could affect attention and concentration, for this purpose we used the numerical rating scales and included patients with a score ≤ 4. We also assessed delayed neurocognitive recovery (dNCR) by estimating the drop in MoCA score (ΔMoCA) between the D-1 MoCA (baseline) and follow-up intervention after two days (D+2). A patient was considered with a delayed neurocognitive recovery (dNCR +) if ΔMoCA > 2 (Supplemental Material).

### Automatic Alpha Band Power Estimation and Propofol TCI at SEF_95_ 8-13 Hz

The EEG signal was collected from EEG monitor in an .edf format, then processed using Matlab R2018a. The variables were extracted within a 5 min long time window where the spectral edge frequency (SEF) remained in a (Berger et al., 2018) Hz range (stable SEF). We recall that the SEF_95_ corresponds to the frequency below which 95% of the signal total power is found. This index reflects the depth of the general anesthesia, such that an SEF_95_ in 8–13 Hz ensures that patient sedation is appropriate to generate an alpha rhythm. On the contrary, a SEF_95_ outside the stable region might be the sign of burst suppression (deep sedation) or beta activity (light sedation). We used an automatic procedure to detect 5 min long 8–13 Hz SEF_95_ region in the signal (details are provided in the Supplementary Section).

### Statistical Analysis

#### Statistical Modeling of Cognitive Decline

We evaluated the risk of presenting a cognitive decline based on age, the combination of Propofol TCI and AP, and all variables considered. Statistical analysis focused on a time window where the SEF_95_ was stable between 8 and 13Hz. Patient CD were labeled using 1 for CD+ (Low MoCA) and 0 otherwise. This binary outcome was modeled as a random variable drawn from a binomial distribution: *CD* ~ *Binomial*(1, *p*), where using the age we had:

(1) $$\begin{array}{l} logit\left(p\right)=\beta_{0}+\beta_{1}\cdot Age.\;\left(AGE\right) \end{array}$$

Based on TCI and AP, we had:

(2) $$\begin{array}{l} logit\left(p\right)=\beta_{0}+\beta_{1}\cdot TCI+\beta_{2}\cdot AP.\;\left(HELP\right) \end{array}$$

For better readability, we have chosen to assign a score from 0 to 100 (%) to the HELP model for each patient reflecting the probability of experiencing cognitive decline.

Finally, to investigate whether or not the three variables together bring complementary information in predicting CD, we extended HELP to include the age such that:

(3) $$\begin{array}{l} logit\left(p\right)=\beta_{0}+\beta_{1}\cdot TCI+\beta_{2}\cdot AP+\beta_{3}\cdot Age.\;\left(HELP2\right) \end{array}$$

The intercept β_0_ as well as coefficients (β_1_, β_2_, β_3_) were optimized numerically based on the data. Models HELP, AGE and HELP2 where compared and evaluated based on the log likelihood. Models were first compared with regard to the data-fit using log likelihood ratio tests. To account for the risk of overfitting, we then compared models based on information criteria penalizing for model complexity in terms of numbers of parameters. We reported the Akaike information criterion (AIC) and Bayesian information criterion (BIC). To explore the data-fit at the level of medical decision-making, we considered receiver operating characteristic (ROC) curves and their areas under the curve (AUC) for each of the three models.

#### Statistical Inference

Estimating the relative impact of the TCI and AP variables on the HELP score, we computed for each variable, based on the probability from HELP, the marginal effect (ME) $m_{TCI}=\frac{\partial p\left(CD|TCI,AP\right)}{\partial TCI}$ and $m_{AP}=\frac{\partial p\left(CD|TCI,AP\right)}{\partial AP}$, that we reported by the mean, standard deviation and confidence interval (CI) of the mean (Thomas J. Leeper 2018, margins: Marginal Effects for Model Objects. R package version 0.3.23.). We can remark that TCI (resp. AP) ME for the logistic model HELP is proportional to coefficient β_1_ (resp. β_2_), thus for instance a null β_1_ (null hypothesis) will translate into a null ME (*m*_*TCI*_ = 0). Finally, we investigated the average ME of one variable conditioned to a specific value of the other one. Such metric assesses the importance of one variable in predicting CD given the value of the second variable. This conditional ME was shown as a graph of the means and standard error.

#### Statistical Analysis

The description of qualitative data was given in percentage while quantitative ones were reported as median and interquartile range (IQR). Patient characteristics were compared between CD+ and CD– groups using a Mann–Whiney test for quantitative variables and a Chi^2^-test for qualitative variables. The significance level for all statistic tests was α = 0.05. Statistical analyses were performed using R version 4.0.

## Results

### Patients

Between November 2018 and May 2019, a total of 56 patients were selected to evaluate the relationship between the per-operative alpha-band, sedation levels during a Propofol based general anesthesia and a pre-operative MoCA psychometric testing. Of these, 14 (25%) did not meet one of the three following conditions: agreed for D-1 MoCA evaluation (4 patients, 7%), had a collected EEG signal (2 missing, 4%) and had a stable SEF_95_ (8 patients, 14%, see Supplementary Flowchart Figure). Forty-two patients were therefore analyzed as having a preoperative cognitive assessment and a stable intraoperative EEG period at SEF_95_ 8–13 Hz (74% female, age = 59.4 ± 18.8 yrs.). 40% (17 patients) had CD+ (MoCA pre-operative <25 points). Fourteen (33%) and 28 (67%) have been admitted for a neuroradiology procedures and orthopedic surgeries, respectively. These patients Propofol TCI and AP were 3.4 [3.0, 3.5] μg/ml and 6.37 ± 3.9 dB, respectively. The median time of surgery was 2.6 h [2.0, 3.3] (Table 1).

**Table 1:** Characteristic population and difference between CD+ and CD- patients (C.V., cardiovascular).

**Variable**	**All (*n* = 42)**	**CD– (*n* = 25)**	**CD+ (*n* = 17)**	***p***
Age (yr)	59.4 ± 18.8	50.48 ± 17.4	72.47 ± 12.2	<0.001
Female	31 (73.8%)	20 (80.0%)	11 (64.7%)	0.268
Orthopedic	28 (67%)	16 (57%)	12 (43%)	0.505
Neuroradiology	14 (33%)	10 (71%)	4 (29%)	
Intervention dura- tion (hours)	2.6[2; 3.3]	2.5[2; 3.1]	3[2; 3.3]	0.533
**Comorbidities**				
MoCA pre	25 [22; 27]	26.5 [25; 28]	20 [14; 23]	<0.001
C.V. Risk Factors	27 (65.9%)	14 (58.3%)	13 (76.5%)	0.227
C.V. History	7 (17.1%)	2 (8.3%)	5 (29.4%)	0.077
Cardiovascular treatments	21 (51.22%)	9 (37.50%)	12 (70.6%)	0.036
Reference MAP (mmHg)	100 [90; 105]	100 [83; 105]	100 [93; 105]	0.365
Neuro. History	5 (12.2%)	4 (16.7%)	1 (5.9%)	0.298
Depression (treated)	6 (14.63%)	5 (20.83%)	1 (5.9%)	0.182
Psychiatric treat- ments	8 (19.5%)	6 (25.0%)	2 (11.8%)	0.292
**Per-operative**				
MAP (mmHg) at SEF95: 8-13 Hz	80 [75; 85]	85 [78; 85]	78 [75; 85]	0.255
T(C°) at SEF95: 8-13 Hz	36.3 [36; 36.6]	36.2 [36; 36.4]	36.4 [36.2; 36.8]	0.423
TCI (μg/ml) at SEF95:8–13 Hz	3.25 [3.0; 3.50]	3.5 [3.0; 3.9]	3 [2.50; 3.0]	<0.001
AP (dB) at SEF95: 8–13 Hz	7.56 ± 3.16	8.88 ± 2.99	5.62 ± 2.34	<0.001
HELP Score (%) at SEF95: 8–13 Hz	35.2 [10.5; 70.4]	10.8 [2.2; 36.9]	71.9 [45.8; 91.5]	<0.001
Cumul. IES (s) (total time)	210 [64; 408]	132 [14; 346]	267 [184; 428]	0.151

### Difference Between CD+ and CD- Patients

CD+ patients were significantly older (72 ± 12 yrs. vs. 50 ± 17, *p* < 0.001), and had neither significantly more cardiovascular nor neurological risk factors (Table 1). Interestingly, CD+ patients had significantly more anti- hypertensive treatments (71 vs. 38%, *p* = 0.036). No significant difference between women and men distribution could be found. The type of surgery was not significantly different between the two groups. Concerning the intra-operative variables, we found that the alpha-band AP was significantly lower in the CD+ group (5.6 ± 2.3 vs. 8.9 ± 3.0 dB, *p* < 0.001). Additionally, these patients required a significantly lower Propofol TCI (3 [2.6, 3.5] μg/ml vs. 3.5 [3, 3.6] μg/ml, *p* < 0.01) to achieve the same anesthetic depth (SEF95: 8–13 Hz). Interestingly, the cumulative time spend in iso-electrical suppression (burst suppression) state was statistically similar between CD + and CD- patients (267 [184; 428] seconds vs. 132 [14; 346] seconds, *p* = 0.151), and was thus poorly associated with CD (AUC = 0.63).

### Model Selection to Predict Occurrence of CD

**Figure 1:**
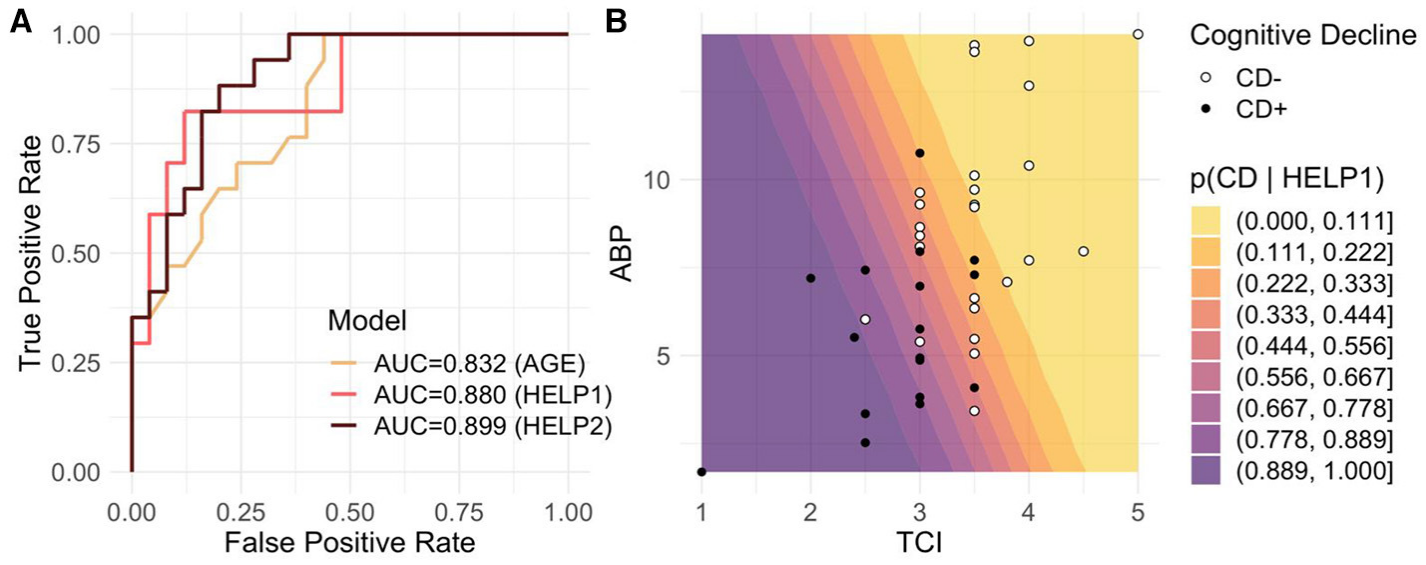
Predicting CD from TCI and AP. We assessed CD prediction based on 3 models including the age (AGE), TCI and AP (HELP1), and the three variables altogether (HELP2). **(A)** We reported logistic based classification results for the three models in a form of ROC curves with their associated AUC, which were 0.832, 0.880, and 0.899 for AGE, HELP1 and HELP2, respectively. Despite better AUC for HELP2, model comparison showed that (HELP2) does not bring significantly more information than HELP (*p* = 0.032 vs. 0.064, likelihood ratio test). We therefore selected HELP1 to model the risk of CD occurrence. **(B)** We investigated the relationship between AP (y-axis), Propofol TCI (a-axis), preoperative MoCA (circles) and the probability to present CD estimated by HELP1 (color gradient). CD+ and CD– patients are depicted by black circles and white circles, respectively. Cold, purple colors indicated high probability to present CD. One can readily see that low MOCA scores were associated with lower TCI and lower alpha power. Some cases are discussed in Figure 4 concerning patients with MoCA and HELP mismatch [white circles in a cold zone, TCI 2.5 μ*g*/*ml*, AP at 6 db (Figure 4C) and black circles in a warmer zone, TCI 3 μ*g*/*ml* and alpha band at 10 db (Figure 4D)].

To evaluate AP, TCI, and age for predicting CD, we benchmarked three logistic models based on the age (AGE), TCI+AP (HELP), and the all three variables together (HELP2). The ROC AUC for the AGE was 0.83, then 0.88 for HELP and 0.90 for HELP2 (see Figure 1A). We found that compared to AGE, HELP significantly better fitted the data than HELP2 (*p* = 0.032 vs. 0.064, likelihood ratio test). Additionally, the AIC (resp. BIC) dropped from 43.7 (47.25) for AGE to 40.84 (46.05) and 39.42 (46.37) for HELP and HELP2, respectively, suggesting that the HELP model was the best trade-off between prediction score and the number of parameters. In summary, the model HELP, including TCI and AP, better predicted CD than age and was more parsimonious than HELP2. Furthermore, at this stage adding age to TCI and AP did not add obvious information to predict CD. Therefore, we focused on the HELP model. We, hence, extrapolated the probability of CD for TCI ranging from 1 to 7 μg/ml and AP from 1 to 15 dB (see Figure 1B). The CD+ and CD- regions are separated by the linear boundary such that a patient is classified as CD + if $TCI<4-\;\frac{1.3}{10}AP.$

### Effect of TCI and AP on Probability of CD

**Figure 2:**
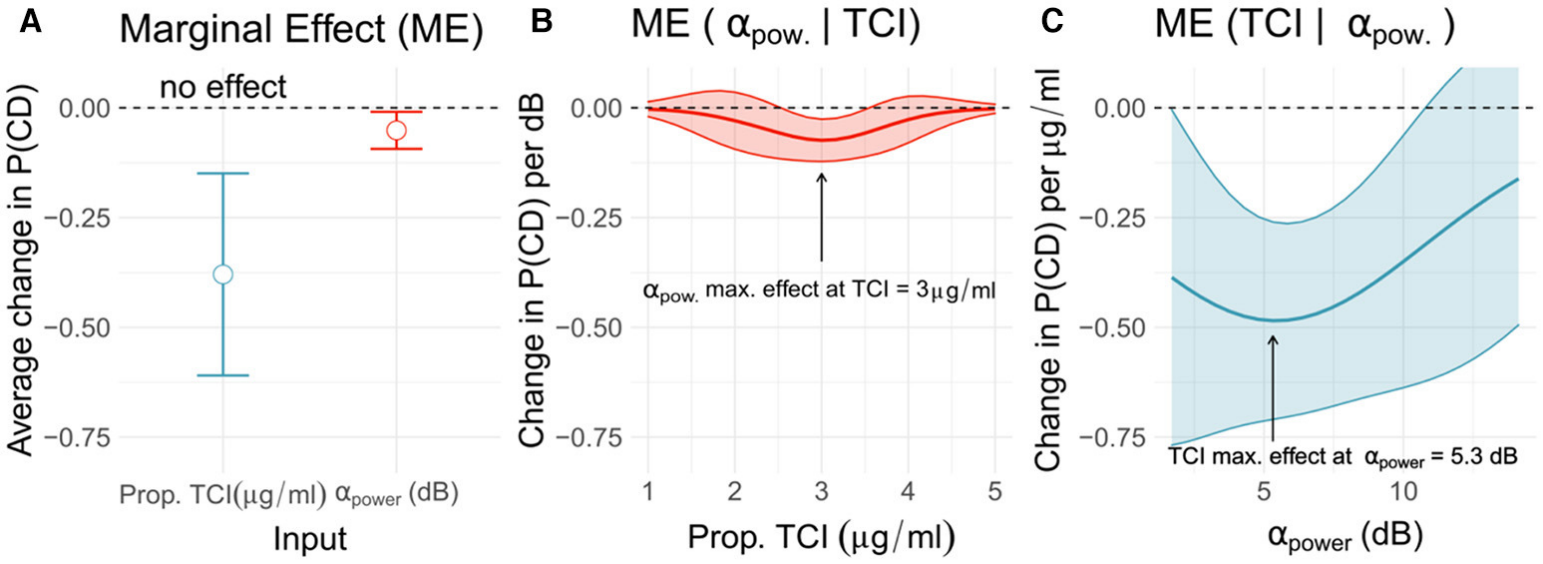
Marginal effects of TCI and alpha power of CD probability. The present triptych shows effect of TCI and AP on the patient's probability to be CD, estimated from the HELP model. On the panel **(A)**, marginal effect means (circle) and standard deviation bar errors are reported for TCI (red) and AP (blue). The two variables contribute to predict the onset of CD among patients, although TCI appears to more strongly affect CD probability compared to AP. Panels **(B,C)** show conditional marginal effects reflecting change in one variable effect relatively to the other variable values. The AP effects is the most important at a TCI of 3 μ*g*/*ml* **(B)**, while TCI strongly impact prediction for an AP of 5.5 dB. Consequently, effect of AP in predicting CD is not homogeneous as TCI changes, with a maximal effect near 3 μ*g*/*ml*.

We were then interested in describing relative effects the TCI and AP variables had on CD probability using the HELP model. We measured each variable's contribution by reporting average marginal effects. We found that the Propofol TCI had a greater impact on CD probability (ME −0.38 ± 0.24, CI = [−0.45, −0.30], *p* = 0.0012) compared to AP (−0.05 ± 0.03, CI = [−0.06, −0.04], *p* = 0.0172). These findings are coherent since TCI modulates AP, however they also reveal the additional effect of these two variables (see Figure 2A). We further analyzed how the average ME of one variable was conditioned by the second. Such analysis aims to explore the impact one specific variable had on the prediction effect of the second one. We found that AP effect on CD probability was small for TCI ranging from 1 to 2 μ*g*/*ml* and from 4 to 5 μ*g*/*ml*, however contribution of AP in predicting CD was the most important at a TCI of 3 μ*g*/*ml* (see Figure 2B). Such local minimum of conditional ME reveals a range of TCI where AP effect increases up to 44% above the average ME (average: −0.051, CI = [−0.062, −0.041] vs. local min.: −0.074 CI = [−0.081, −0.067]). On the other hand, the Propofol TCI conditional ME remained below −25% per unit of TCI for almost all AP-values and was the most important at AP = 5.5 dB (see Figure 2C), showing that weak and strong AP discriminated patients likely to have a CD. In summary, TCI and AP revealed additive and complementary information on CD.

**Figure 3:**
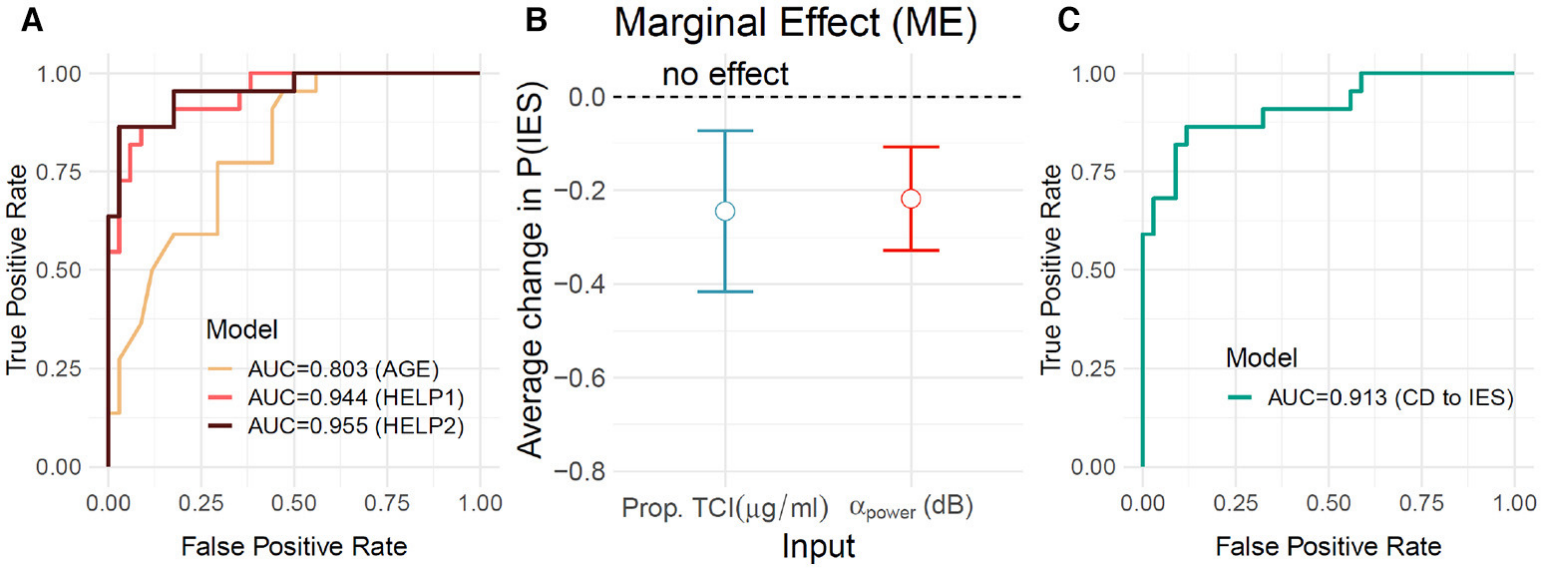
Generalization testing on burst suppression data. To probe the generalization of the proposed model beyond the given sample we considered the observational data from Touchard et al. (2019) in which burst suppression was studied as a proxy for cognitive decline. The dataset comprised 56 patients sedated with Propofol. None of these patients was included in the previous analyses. In a first step **(A)**, we applied the models as defined in the previous analyses (Figures 1, 2) to predict the occurrence of IES during the induction period. HELP1 (TCI + ABP) predicted IES (AUC=0.94, orange) better than AGE (AUC=0.80, yellow). Prediction did not increase significantly for HELP2 (TCI + ABP + Age) as compared to HELP1. We then inspected the HELP1 (IES) model **(B)**. As in previous analyses, TCI (blue) and ABP (red) showed complementary average marginal effects (mean±SE: −0.25±0.09, −0.22±0.06 for TCI and ABP, respectively). We then directly quantified generalization performance **(C)** of the HELP1 (CD) on the IES dataset (AUC=0.91). The results suggest that the proposed HELP model generalizes beyond the observed sample and captures cognitive and physiological factors related to postoperative outcome.

We further proceeded by evaluating the generalization performance of the proposed model of CD (Figure 3) to distinct but related data on iso-electrical suppressions (IES), the flat component of the burst-suppression. We considered 56 patients from a previous observational study on the relationship between ABP, TCI and IES (Touchard et al., 2019) in which IES was investigated as a proxy for CD. Results suggest that our proposed model generalizes well-beyond the current dataset and captures physiological variance linked to cognitive decline and drug sensitivity.

## Discussion

In the present work, we have found that a lower Propofol requirement (TCI) and a weaker frontal alpha-band power (AP) observed during a general anesthesia (SEF_95_ in 8–13 Hz) could be used to reveal pre-existing cognitive decline. These two variables performed, in our patient group, better than the chronological age. The effect of AP on the patient probability to have CD was the most prominent at TCI at 3 μ*g*/*ml*, and, in a more interesting way, TCI had a much greater effect than alpha band on the likelihood of pre-operative cognitive decline. We further confirmed these finding via HELP model generalization analysis performed on a secondary data set of 56 patients. These results highlight the importance of interpreting cognitive EEG biomarkers (AP) accounting for the hypnotic concentration (TCI). Such information, in daily practice, could help clinician to access patient's biological brain age and then a possible fragility. As more and more studies underline the influence of the preoperative cognitive state on the post-operative outcome (Brown et al., 2016; Culley et al., 2017). Taking advantage of general anesthesia to evaluate brain responses to a stress and identify a given vulnerability seems to be an opportunity to be seized, especially as growing number of anesthesia are performed every year and mostly concerns middle age and elderly patients.

Patients with higher preoperative MoCA scores required on average a higher TCI to obtain stable intra-operative EEG-signals within an 8–13 Hz SEF_95_. These findings are consistent with Laalou and colleagues who reported that TCI was directly influenced by pre-operative cognitive performances (Laalou et al., 2010). In our cohort, the Propofol requirement represented by the TCI set to achieve a SEF_95_ between 8 and 13 Hz has a major effect on the probability of experiencing cognitive decline. When TCI is either very low or high, it is alone sufficient to classify whether the patient is at risk or not. All of our patients with required TCI below 2.5 μ*g*/*ml* had preoperative cognitive decline and those requiring more than 4 μ*g*/*ml* of Propofol had a good cognitive health. Despite AP playing a minor role in predicting CD in this situation (Figure 2B), patients with low TCI had nonetheless a low AP too (Figure 1B).

The association between low alpha-band and cognitive impairment are consistent with recent work from Koch and colleagues (Koch et al., 2019). The analysis of the average AP effect (average marginal effect Figure 2A) showed a significant association with CD. In our study, conditional analysis exposed an inverse U-shaped behavior where AP plays a more important role in predicting CD when the TCI values range from 2.5 to 4 μ*g*/*ml* with maximal impact at TCI 3 μ*g*/*ml*. Within such range, it is in general not clear for the practitioner whether the patient is fragile or not, which may explain why alpha power helps to further distinguish CD from non-CD patients.

In summary, once a SEF_95_-defined stable anesthesia is achieved, the associated TCI is collected. If the TCI is high (> 4 μ*g*/*ml*) or low (<2.5 μ*g*/*ml*), then it is sufficient to stratify the risk, otherwise we have to evaluate the alpha-band power. There, the lower the alpha-band power, the greater the probability of cognitive decline. Nevertheless, one should keep in mind that AP is influenced by TCI (Purdon et al., 2015; Cartailler et al., 2019). Indeed, a patient with good cognitive performance might have a low AP in the case where the TCI would have been high enough. In the light of our results, establishing relationships between AP, age and cognition without considering the Propofol concentration could exposes one to a lack of precision at best and to risk stratification errors at worst.

Despite important effect sizes, the number of patients was small. We attempted to address this issue by generalizing the HELP model to the dataset from our previous study (Touchard et al., 2019) and found that using iso-electrical suppression was linked to cognitive fragility. Still, as explained in detail by Wildes and colleagues, burst-suppression interpretation is protocol-dependent, hence might not always be the best indicator of CD. This is perfectly well illustrated by the poor association we found between CD and IES in the new set of 42 patients for whom IES was proactively avoided. Another limitation concerns the population-based pharmacokinetics estimation of TCI within the TIVA framework. These models could be ill estimated for frail patients, especially as TIVA models, such as Schnider's, do not adapt for either variations in cardiac output or clearance that often occur among fragile patients. Therefore, in such situation, TCI values predicted by the model would not be true and hence might fail to reflect a specific cognitive fragility.

The MoCA psychometric test also possesses intrinsic limitations although it is a fair trade-off between a short test and one evaluating several cognitive functions. It is particularly interesting in detecting mild cognitive impairments which are considered to be pre-clinical signs of Alzheimer dementia (Nasreddine et al., 2005). However, the trained human resources required to administer MoCA is a limiting condition often preventing its routine use in perioperative period.

**Figure 4:**
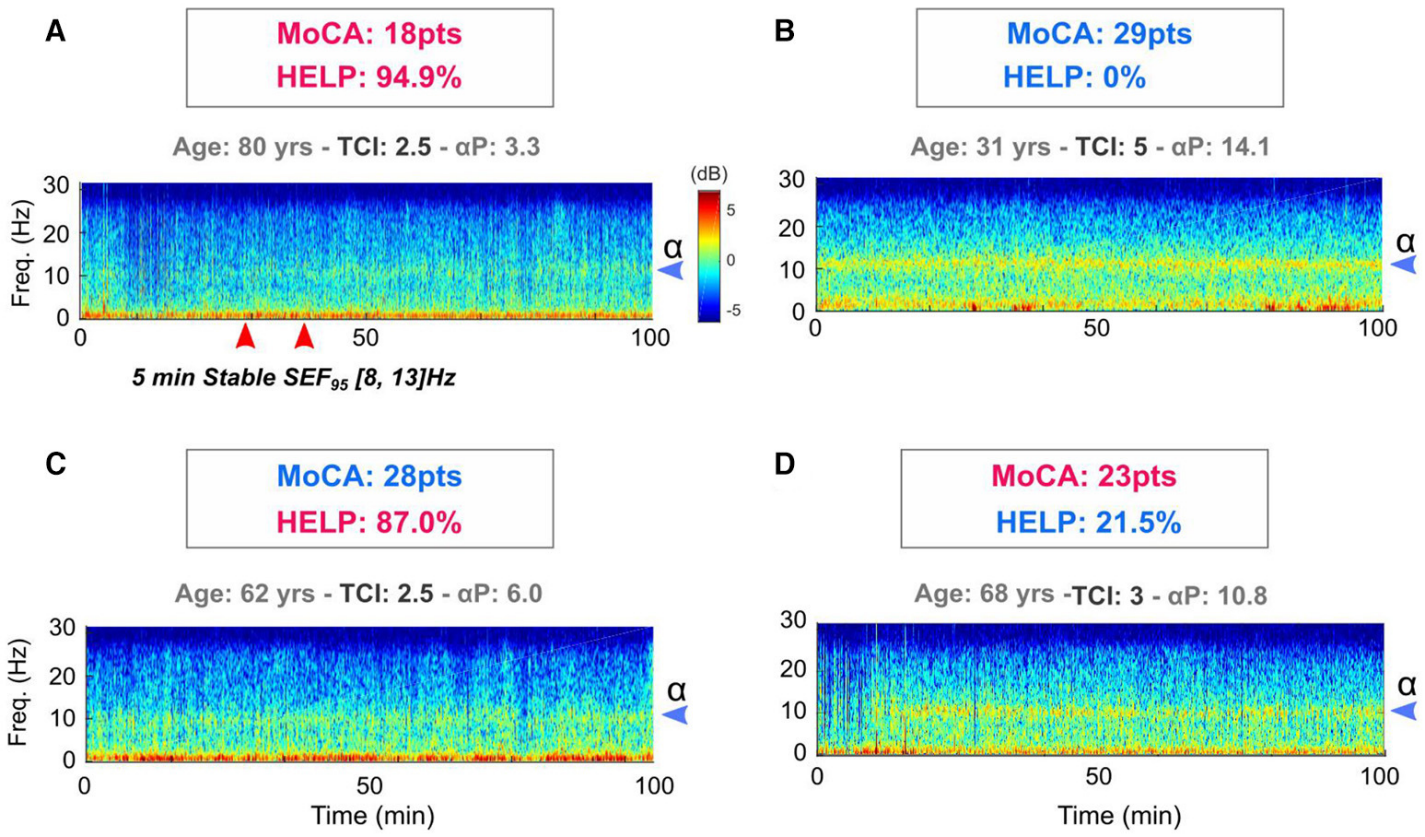
The potential contribution of intra operative HELP model in relation to preoperative MoCA in the detection of cognitive decline of anesthetized patients. For two subjects **(A,B)**, there are classification concordance between MoCA and HELP model. These are deliberately caricatural situations in order to clearly differentiate the intraoperative characteristics that exist between a subject with a low MoCA **(A)** and young subject without cognitive impairment **(B)**. In these situations, neither the HELP model, nor the pre-operative cognitive evaluation provides information in relation to the date of birth. An 80-year-old subject **(A)** is at risk of cognitive complications and a 32-year-old patient **(B)** is not. Things could be more interesting in MoCA and HELP mismatch situations for middle-aged patients **(C,D)**. If subject C presents a normal pre- operative MoCA and is relatively young, it is striking to observe his weak peroperative variables. In fact, this 62-year-old patient suffered a ruptured aneurysm at age 42 with a major subarachnoid hemorrhage that warranted a lengthy intensive care unit hospitalization and described memory and attention complaints later in life. HELP model could appear more robust to detect past and so maybe future brain suffering. On the contrary, the patient D (68 years old woman) presents a robust HELP score derived from the model, discordant with a low preoperative MoCA. The clinical history of this patient with difficult socioeconomic conditions shows chronic anxiety disorders and a marked apprehension of the upcoming surgery. Considering these two situations, the intraoperative variable could be used to help stratify perioperative cognitive risk when MoCA is taken in default, particularly for middle-aged patients (50 to 70 years old).

Additionally, false positive CD diagnosis might occur for several reasons including stress or pain (Figure 4). On the contrary, patients with significant cognitive reserve might pass the neuropsychological screening, yet still, show a cognitive fragility that could manifest itself as a post-operative cognitive dysfunction, such as delirium. For these reasons, EEG and hypnotic concentration are physiological and biological measures that could be more appropriate to detect patients at risk of developing neurocognitive disorders.

## Conclusion

The power of the alpha band combined with the concentration of Propofol required to achieve unconsciousness could be an effective, objective and reliable tool to detect cognitive decline in the preoperative period. This approach promises to minimize human bias using intra-operative data already collected in routine care. Future studies will need to be conducted to test this type of indirect cognitive assessment by intra operative data modeling (HELP model) for predicting post-operative chronic cognitive disorder by comparing or implementing it to preoperative neuro-cognitive scales. This approach might allow anesthesiologists to play an active role in detecting, predicting and preventing neurocognitive disorders.

## Data Availability Statement

The original contributions presented in the study are included in the article/Supplementary Material, further inquiries can be directed to the corresponding author/s.

## Ethics Statement

The studies involving human participants were reviewed and approved by the SRLF (Société de réanimation de la langue française) Ethics Advisory Committee 11–356. Written informed consent for participation was not required for this study in accordance with the national legislation and the institutional requirements.

## Author Contributions

CT and JC: study design, data analysis, patient recruitment, data collection, and redaction of the manuscript. CL and JS: patient recruitment and data collection. DS: data analysis. EM: patient recruitment and data collection. JJ and JM: data collection. EG: data analysis. DE: data analysis and manuscript redaction. FV: study design, patient recruitment, data collection, and manuscript redaction. All authors contributed to the article and approved the submitted version.

## Conflict of Interest

The authors declare that the research was conducted in the absence of any commercial or financial relationships that could be construed as a potential conflict of interest.
