# Supplementary material for: Propofol Requirement and EEG Alpha Band Power During General Anesthesia Provide Complementary Views on Preoperative Cognitive Decline
Source: Front Aging Neurosci. 2020 Nov 27;12:593320. doi: 10.3389/fnagi.2020.593320 (PMC7729157; doi:10.3389/fnagi.2020.593320)
Supplement: Supplementary file 1 [file Image_1.pdf]

Propofol Requirement and EEG Alpha Band Power During General Anesthesia Provide Complementary Views on Preoperative Cognitive Decline

Supplementary Material

Cyril Touchard[1*], Jérôme Cartailler[1,2*], Charlotte Levé[1], José Serrano[1], David Sabbagh[3], Elsa Manquat[1], Jona Joachim[1], Joaquim Mateo[1], Etienne Gayat[1,2], Denis Engemann[3,4] and Fabrice Vallée[1,2,3]

[1] Department of Anesthesiology and Intensive Care, Lariboisière – Saint Louis Hospitals, Paris, France, [2] Inserm, UMRS-942, Paris Diderot University, Paris, France, [3] Université Paris-Saclay, Inria, CEA Palaiseau, France, [4] Department of Neurology, Max Planck Institute for Human Cognitive and Brain Sciences, Leipzig, Germany

Corresponding authors:

Cyril Touchard, cyriltouchard@hotmail.fr and Jerome Cartailler cartailler.jerome@gmail.com.

** These authors contributed equally.*

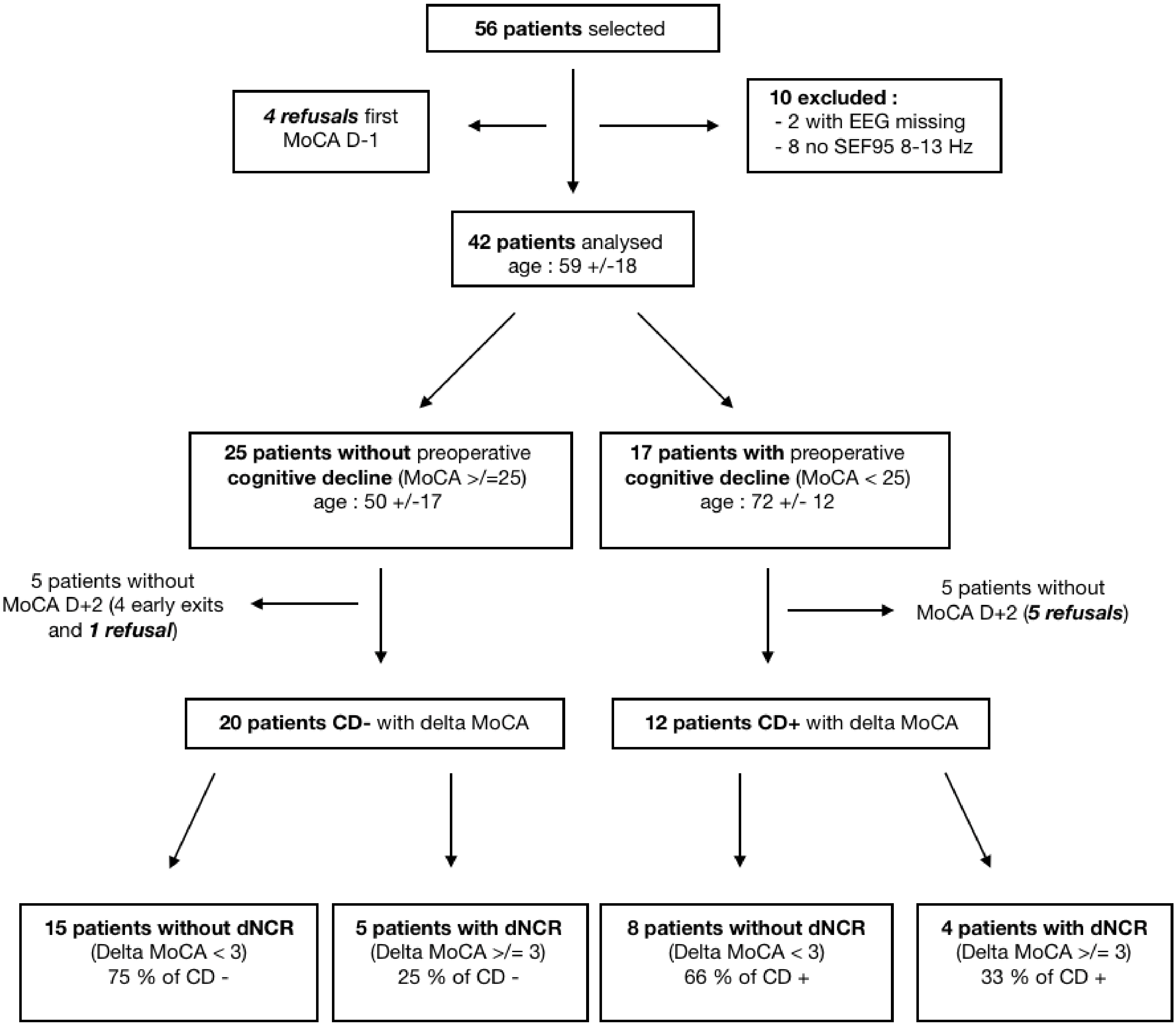

Figure 1: Flowchart

Delayed neuro cognitive recovery (dNCR)

Among the first 42 included patients, 6 (14 %) refused the D+2 MoCA evaluation, while 4 (10 %) could not be evaluated as a result of an early departure. In the end, 32 patients had a MoCA evaluation at both D-1 and D+2 and 28% of them (9 patients) were dNCR+ (delta MoCA > 2pts).

We turned our attention toward the interplay between CD, AP, TCI and dNCR events, the latter evaluated based on the delta MoCA estimated from MoCA drop between D-1 and D+2. Only 32 (57%) delta MoCA were established based on the 56 patients initially included, exposing concerns about deploying such methodology in daily practice. A total of 9 patients out of 32 (28%) were dNCR+, almost similarly distributed between CD+/- patient: 5 (25%) in the CD- vs 4 (33%) in the CD+. In addition, we found no differences in age (p=0.418), AP (p=0.967) or TCI (p=0.23).

Characteristics of patients refusing MoCA psychometric testing

We describe characteristics of patients refusing either D-1 or D+2 MoCA evaluation. Ten patients among 56 (18%) refused to be screened with the MoCA method either at D-1 (n=4, 7%) or D+2 (n=6, 11%). While early MoCA refusals are responsible for a decrease in the number of patients included in the study, refusals at D+2 induce a bias in the dNCR interpretation. Interesting, among CD+ patients, 5 (29%) refused to undergo a D+2 MoCA compared to only 1(4%) among CD- (p=0.03). Moreover, among patient refusing D+2 evaluation, all had a low MoCA at D-1. They were significantly older than patients included in the model (73[70; 79] yo. vs 59.5[37.5; 72] yo., p=0.026), had significantly lower D-1 MoCA scores (21[15; 23] vs 25[23, 27], p<0.006), a lower TCI (3 [2.6; 3] vs. 3.5 [3; 3.5] $\mu g/ml$, p=0.034) yet not significant difference in AP (7.1[5.8; 7.3]dB vs 7.7[5.4; 9.7], p=0.140). In summary, patient likely to present cognitive decline are more inclined to refuse a psychometric testing.

Complementary information concerning signal processing

The estimation of the SEF_{95} was performed by computing the signal total power spectral density (PSD), from at 0.5Hz to 25Hz, for the left (F7, Fp1) and right (F8, Fp2) couples of frontal electrodes. After the identification of the SEF_{95} for each side, the averaged was taken to obtain a single SEF_{95} time series. Then, the SEF_{95} time series was smoothed-out using a rolling average filter over a 45 seconds long window to remove noise related fluctuations. Finally, to prevent GA induction instabilities to corrupt the analysis, we have added the following constraints: the 5 min time window with a stable SEF_{95} had to be at least 25 min

following GA initiation, the MAP and the temperature had to be above 70 mmHg and 36°C respectively.

The alpha-band power (AP) is referring here to the difference between the PSD maximum value (in decibels) estimated within 8-13Hz and the PSD background as in Hight and colleagues paper [1]. The PSD was computed from 15s long overlapping windows, generated every seconds and applying a Hann's function to window the EEG signal. The PSD background is the spectrum curve decrease (in dB) fitted with the function $ax + b + c/x$ using least absolute residual method and ignoring the ranges 0-3Hz (slow signal) and 8-13Hz (alpha peak). We note that since curare were administered to patients, and signal picked from stable SEF periods, the contribution of signal's artifacts was negligible. Nevertheless, we added a naïve threshold detector such that if the absolute value of the signal amplitude was above 80μV, a 1s wide portion of it was rejected. AP from Fp1 and Fp2 were computed separately then averaged.

Finally, for the stable SEF_{95} periods, we determined the average Propofol TCI from doses during the surgery. The total time spent in burst suppressions, evaluating durations where the EEG signal had an absolute amplitude below 5μV, was obtained from Sedline monitors.

Reference

[1]. D. Hight, L. J. Voss, P. S. Garcia, and J. Sleigh, "Changes in Alpha Frequency and Power of the Electroencephalogram during Volatile-Based General Anesthesia," Front. Syst. Neurosci., vol. 11, 2017, doi: 10.3389/fnsys.2017.00036.
